# Supplementary material for: Impact of COVID-19-Related Social Isolation on Behavioral Outcomes in Young Adults Residing in Northern Italy
Source: Int J Environ Res Public Health. 2022 Dec 8;19(24):16496. doi: 10.3390/ijerph192416496 (PMC9779507; doi:10.3390/ijerph192416496)
Supplement: Supplementary file 1 [file ijerph-19-16496-s001.zip › ijerph-2022696-supplementary.pdf]

Supplementary Materials

Table S1. **Complete ASR scores at the baseline and follow-up visit.** ASR scores (median, 1st and 3rd quartile) obtained baseline and follow-up and the differences estimated between the two time-points for 65 PHIME participants. P-values are estimated through the sign test.

ASR score	Baseline	Follow-up	$ASR_{pre-post}$	p
Anxious Depressed	58.0 (53.0, 65.0)	58.0 (52.0, 63.2)	0.0 (-3.2, 3.0)	1.000
Withdrawn	55.0 (51.0, 65.0)	55.0 (50.0, 63.0)	-1.0 (-4.0, 3.0)	0.108
Somatic Complaints	56.0 (51.0, 62.0)	54.0 (51.0, 58.0)	0.0 (-4.0, 2.0)	0.480
Thought Problems	51.5 (50.0, 55.8)	53.5 (51.0, 58.2)	1.0 (-1.0, 4.0)	0.049
Attention Problems	54.0 (51.0, 58.0)	55.0 (51.0, 58.2)	0.0 (-2.0, 3.0)	1.000
Rule Breaking Behavior	51.0 (50.0, 53.0)	51.0 (50.0, 52.0)	0.0 (-1.0, 0.0)	0.188
Aggressive Behavior	52.5 (50.0, 57.0)	53.0 (51.0, 58.0)	0.0 (-2.0, 3.0)	0.784
Internalizing Problems	59.0 (52.0, 66.0)	58.0 (51.0, 64.0)	-2.0 (-6.2, 4.0)	0.124
Externalizing Problems	51.5 (42.8, 55.0)	50.0 (43.8, 54.2)	0.0 (-5.0, 4.0)	1.000
Total Problems	52.5 (48.0, 60.0)	53.0 (46.8, 58.0)	0.0 (-4.2, 3.0)	0.590

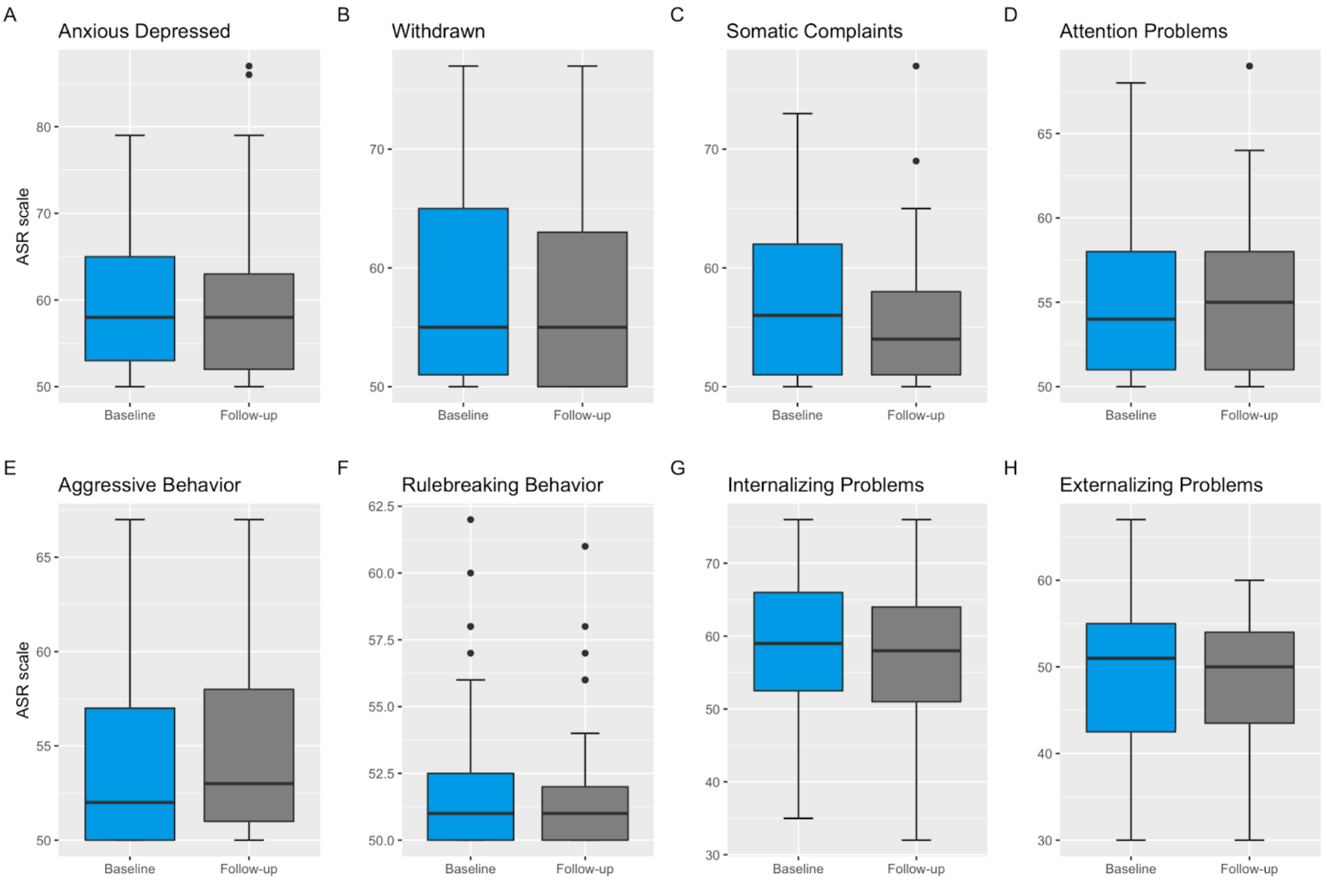

Figure S1. Differences in all ASR score between baseline and follow up. The boxplot shows the scores obtained in six clinical scales measured by the ASR and in "Internalizing " and "Externalizing" problems scale of the questionnaire in the two time points considered: baseline visit (blue box) and follow up visit (gray box).

Table S2. **Interaction between socio-demographic variables and ASR scales.** Relationship between the sociodemographic variables (sex age, SES and IQ) of the sample and the psychopathological outcomes at the follow-up visit.

	Anxious Depressed			Withdrawn			Somatic Complaints			Thought Problems			Attention Problems		
Predictors	*Estimates*	*CI*	*p*	*Estimates*	*CI*	*p*	*Estimates*	*CI*	*p*	*Estimates*	*CI*	*p*	*Estimates*	*CI*	*p*
High vs Low SI	0.62	-2.91 – 4.16	0.725	-0.32	-4.43 – 3.79	0.877	-1.27	-4.51 – 1.98	0.438	0.1	-2.66 – 2.86	0.943	-0.22	-2.85 – 2.40	0.866
Sex: Females vs Males	0.83	-2.34 – 4.00	0.602	-1.04	-4.58 – 2.51	0.559	2.8	-0.11 – 5.72	0.059	-0.23	-2.71 – 2.24	0.852	1.38	-0.98 – 3.73	0.247
SES: Medium vs Low	1.92	-2.06 – 5.90	0.338	-0.1	-4.73 – 4.52	0.964	-0.71	-4.36 – 2.94	0.700	-0.47	-3.58 – 2.63	0.761	-0.75	-3.70 – 2.20	0.613
SES: High vs Low	1.21	-3.38 – 5.80	0.600	-4.21	-9.55 – 1.14	0.120	-1.29	-5.50 – 2.92	0.542	-2.18	-5.76 – 1.40	0.228	0	-3.41 – 3.41	0.999
Age	-0.08	-0.75 – 0.59	0.812	0.11	-0.82 – 1.05	0.809	0.59	-0.02 – 1.21	0.060	0.29	-0.23 – 0.82	0.270	0.31	-0.19 – 0.81	0.214
IQ	0.17	0.01 – 0.34	**0.04**	0.22	0.05 – 0.39	**0.012**	0.06	-0.09 – 0.21	0.460	0.05	-0.07 – 0.18	0.413	0.13	0.00 – 0.25	**0.043**
	**Rulebreaking Behavior**			**Aggressive Behavior**			**Internalizing Problems**			**Externalizing Problems**			**Total Problems**		
High vs Low SI	1.73	-0.03 – 3.48	0.053	1.21	-1.54 – 3.95	0.381	-0.77	-4.73 – 3.19	0.700	2.2	-2.07 – 6.47	0.306	0.6	-2.67 – 3.87	0.714
Sex: Females vs Males	0.48	-1.09 – 2.05	0.542	0.06	-2.40 – 2.53	0.959	0.82	-2.74 – 4.37	0.647	0.25	-3.58 – 4.07	0.898	0.49	-2.44 – 3.42	0.740
SES: Medium vs Low	-1.14	-3.11 – 0.83	0.249	-0.11	-3.20 – 2.98	0.943	2.2	-2.25 – 6.65	0.327	2.7	-2.10 – 7.49	0.265	1	-2.67 – 4.67	0.588
SES: High vs Low	-1.36	-3.63 – 0.92	0.237	0.32	-3.25 – 3.88	0.860	-0.88	-6.01 – 4.26	0.733	2.6	-2.94 – 8.14	0.351	-0.9	-5.14 – 3.34	0.672
Age	0.35	0.02 – 0.68	**0.04**	-0.13	-0.66 – 0.39	0.611	-0.07	-0.82 – 0.69	0.859	-0.19	-1.01 – 0.62	0.634	0.2	-0.42 – 0.83	0.514
IQ	0.04	-0.04 – 0.12	0.342	0.12	-0.00 – 0.25	0.055	0.18	-0.01 – 0.36	0.060	0.13	-0.07 – 0.33	0.189	0.15	0.00 – 0.30	**0.048**

Table S3. Association between time in social isolation and the ASR scales by sex. Relationship between the time spent in social isolation and the psychopathological outcomes at the follow-up visit by sex. Models were adjusted by age, SES and IQ.

	Anxious Depressed diff			Withdrawn diff			Somatic Complaints diff			Thought Problems diff			Attention Problems diff		
Predictors	*Estimates*	*CI*	*p*	*Estimates*	*CI*	*p*	*Estimates*	*CI*	*p*	*Estimates*	*CI*	*p*	*Estimates*	*CI*	*p*
Females with High SI vs Females with Low SI	1.69	-2.90 – 6.28	0.463	-2.11	-6.87 – 2.66	0.377	-2.25	-6.46 – 1.96	0.289	0.01	-3.55 – 3.57	0.996	-1.56	-4.94 – 1.81	0.358
Males with Low SI vs Females with Low SI	-0.08	-3.87 – 3.72	0.968	-0.26	-4.21 – 3.68	0.894	-3.50	-6.98 – -0.02	**0.049**	0.14	-2.80 – 3.09	0.922	-2.32	-5.11 – 0.47	0.101
Males with High SI vs Females with Low SI	-2.73	-10.16 – 4.69	0.464	6.38	-2.50 – 15.26	0.154	2.52	-4.29 – 9.33	0.461	-0.40	-6.21 – 5.42	0.892	3.43	-2.04 – 8.89	0.214
SES: Medium vs Low	1.58	-2.52 – 5.68	0.443	0.29	-4.31 – 4.89	0.900	-0.40	-4.16 – 3.37	0.834	-0.17	-3.34 – 3.00	0.916	-0.33	-3.34 – 2.69	0.829
SES: High vs Low	0.76	-4.02 – 5.53	0.752	-3.65	-8.99 – 1.68	0.174	-0.87	-5.25 – 3.51	0.692	-2.05	-5.79 – 1.69	0.276	0.57	-2.94 – 4.08	0.746

Age	-0.04	-0.73 – 0.64	0.896	-0.03	-0.98 – 0.91	0.941	0.56	-0.07 – 1.19	0.079	0.23	-0.30 – 0.76	0.386	0.27	-0.23 – 0.77	0.287
IQ	0.18	0.01 – 0.34	**0.037**	0.20	0.03 – 0.37	**0.019**	0.05	-0.10 – 0.20	0.492	0.05	-0.08 – 0.18	0.471	0.12	-0.00 – 0.24	0.050
	**Rulebreaking Behavior**			**Aggressive Behavior**			**Internalizing Problems**			**Externalizing Problems**			**Total Problems**		
Females with High SI vs Females with Low SI	0.12	-2.06 – 2.30	0.910	0.80	-2.78 – 4.37	0.656	-1.06	-6.22 – 4.10	0.682	0.86	-4.68 – 6.39	0.757	-0.98	-5.19 – 3.22	0.642
Males with Low SI vs Females with Low SI	-1.61	-3.41 – 0.19	0.078	-0.35	-3.31 – 2.60	0.812	-1.02	-5.29 – 3.24	0.632	-1.19	-5.77 – 3.38	0.603	-1.61	-5.08 – 1.87	0.358
Males with High SI vs Females with Low SI	4.10	0.57 – 7.62	**0.024**	1.05	-4.73 – 6.83	0.717	0.75	-7.60 – 9.10	0.858	3.43	-5.52 – 12.38	0.446	4.05	-2.76 – 10.85	0.238
SES: Medium vs Low	-0.64	-2.59 – 1.31	0.513	0.02	-3.17 – 3.21	0.990	2.29	-2.32 – 6.90	0.323	3.12	-1.82 – 8.06	0.211	1.50	-2.26 – 5.25	0.427
SES: High vs Low	-0.68	-2.94 – 1.59	0.553	0.49	-3.23 – 4.21	0.793	-0.75	-6.12 – 4.61	0.779	3.17	-2.58 – 8.93	0.274	-0.23	-4.60 – 4.14	0.917
Age	0.30	-0.03 – 0.62	0.072	-0.15	-0.68 – 0.39	0.582	-0.08	-0.85 – 0.69	0.842	-0.24	-1.06 – 0.58	0.563	0.15	-0.47 – 0.78	0.630
IQ	0.03	-0.05 – 0.11	0.401	0.12	-0.01 – 0.25	0.060	0.17	-0.01 – 0.36	0.064	0.13	-0.07 – 0.32	0.208	0.15	-0.00 – 0.30	0.057
